# Guillain-Barré Syndrome With Asymmetrical Weakness in a Patient With Charcot-Marie-Tooth Disease Type 1A

**DOI:** 10.7759/cureus.94525

**Published:** 2025-10-14

**Authors:** Kawser Ahmed, Mohammed Elachola, Hasan Alsararatee, Nichola Pugh

**Affiliations:** 1 Acute Internal Medicine, Northampton General Hospital NHS Trust, Northampton, GBR; 2 Internal Medicine, Northampton General Hospital NHS Trust, Northampton, GBR; 3 Acute Medicine, Northampton General Hospital NHS Trust, Northampton, GBR

**Keywords:** acute low back pain, albuminocytological dissociation, atypical gbs, guillain-barré syndrome (gbs), lower limb weakness

## Abstract

A man in his 60s presented with acute, progressive right lower limb weakness, low back pain, and paraesthesia. Neuroimaging ruled out spinal cord and central pathology. Cerebrospinal fluid analysis revealed albumin-cytological dissociation, and nerve conduction studies confirmed Guillain-Barré syndrome (GBS) superimposed on Charcot-Marie-Tooth disease type 1A (CMT1A). There was no preceding infection or ganglioside antibody positivity. His weakness progressed rapidly, resulting in flaccid paralysis of the right leg. He received intravenous immunoglobulin with marked clinical improvement and regained independent mobility by follow-up. This case is notable for its atypical, asymmetrical presentation of GBS in the context of pre-existing hereditary neuropathy, posing diagnostic challenges. It contributes to the limited literature on overlapping GBS and CMT1A and reinforces the importance of early investigation and immunotherapy in non-classical cases. The case highlights that reflex loss and chronic neuropathy should not preclude consideration of acute treatable causes.

## Introduction

Guillain-Barré syndrome (GBS) is a relatively uncommon disorder in the United Kingdom, with an estimated prevalence of 1-2 per 100,000 population annually [1]. It remains one of the most frequent causes of acute bilateral neuromuscular weakness. GBS typically results from an autoimmune response targeting peripheral nerves, often following an upper respiratory tract infection, such as Epstein-Barr virus, cytomegalovirus, or influenza, or a gastrointestinal illness, particularly *Campylobacter jejuni* [2]. There are several recognised variants of GBS, including acute inflammatory demyelinating polyradiculoneuropathy, acute motor axonal neuropathy, and acute motor and sensory axonal neuropathy. A regional variant, Miller Fisher syndrome, is characterised by ophthalmoplegia, ataxia, and areflexia, and is associated with anti-GQ1b antibodies [3]. The clinical features often include paraesthesia, progressive symmetrical lower limb weakness, low back pain, and autonomic dysfunction, such as postural hypotension, arrhythmia (notably bradyarrhythmia), constipation, urinary retention, and cranial nerve involvement [4]. In severe cases, respiratory muscle paralysis is the principal cause of mortality. Notably, fewer than 1% of GBS cases present with unilateral weakness [5].

Examination findings typically include areflexia, sensory deficits, bilateral facial nerve palsy, and occasionally saddle anaesthesia [6]. Cerebrospinal fluid analysis usually reveals albumin-cytological dissociation-elevated protein with normal cell counts. Nerve conduction studies confirm the diagnosis, often demonstrating conduction block and prolonged F-waves [7]. Intravenous immunoglobulin (IVIG) is the first-line treatment, with plasma exchange as an effective alternative. Respiratory function must be monitored closely, and patients should be managed in an intensive care setting if respiratory compromise is suspected [8]. Prognosis is generally favourable; approximately 80% of patients recover without significant residual weakness, though 20% may experience persistent disability, and up to 5% may die despite treatment [9].

This case report highlights a diagnostically challenging scenario where GBS coexists with Charcot-Marie-Tooth disease type 1A (CMT1A), a chronic hereditary demyelinating neuropathy. While GBS involves a rapid onset, immune-mediated nerve damage, CMT1A causes slowly progressive, genetically determined demyelination. The overlapping features can complicate diagnosis, particularly in atypical cases. The presentation of atypical weakness in the context of both conditions is extremely rare, with very few cases reported in the literature.

## Case presentation

A man in his 60s presented to the Emergency Department (ED) with a seven-day history of progressive right lower limb weakness, accompanied by acute low back pain and sensory disturbance, including tingling and numbness. The pain was localised to the lumbar region, described as dull, non-radiating, and rated 7/10 in intensity. It was exacerbated by movement and partially relieved with oral paracetamol. He reported no alarming symptoms such as fever, weight loss, or night sweats, and denied recent trauma, contact with unwell individuals, or bowel or bladder dysfunction.

His medical history was notable for CMT1A, prior cerebrovascular disease (haemorrhagic stroke), testicular malignancy in remission, hypertension, pes cavus, atrial fibrillation (not anticoagulated following a previous episode of upper gastrointestinal bleeding), and osteoarthritis. He had no known drug allergies. His prescribed medications included losartan 25 mg once daily, rosuvastatin 5 mg once daily, and bisoprolol 1.25 mg once daily. He was a non-smoker and consumed alcohol socially.

On initial assessment, the patient was haemodynamically stable. His temperature was 36.8°C, heart rate was 68 beats per minute, blood pressure was 128/76 mmHg, respiratory rate was 17 breaths per minute, and oxygen saturation was 98% on room air. Spinal assessment demonstrated localised tenderness in the lower lumbar region but no gibbus, localised infection, or signs of traumatic injury. Neurological examination of the upper limbs was unremarkable. The lower limbs, however, demonstrated asymmetrical findings: muscle power was 3/5 on the right (using the Medical Research Council (MRC) scale) and 5/5 on the left; tone was reduced on the right side but normal on the left. Reflexes were absent bilaterally due to his previous CMT1A. Spinothalamic tract sensation (pain and temperature) was absent in both limbs, though the dorsal column sign (vibration and proprioception) was preserved (Table 1). The MRC scale was used for muscle power assessment.

**Table 1:** Primary assessment after lower limb examinations. MRC = Medical Research Council; STS = spinothalamic tract sensation; DCS = dorsal column sign

	Power on the MRC scale	Tone	Reflexes	STS	DCS
Right	3/5	Reduced	Absent	Lost	Present
Left	5/5	Normal	Absent	Lost	Present

Following initial clinical assessment in the ED, the patient’s asymmetrical lower limb weakness raised concern for a possible acute spinal cord lesion or cerebrovascular event. A comprehensive set of blood tests was performed to establish a baseline and exclude metabolic, infective, or haematological causes as outlined in Table 2.

**Table 2:** Laboratory results during the diagnostic workup. WCC = white cell count; MCV = mean corpuscular volume; CRP = C-reactive protein; Na = sodium; K = potassium; eGFR = estimated glomerular filtration rate; ALP = alkaline phosphatase; ALT = alanine aminotransferase; APTT = activated partial thromboplastin time; INR = international normalised ratio

Test	Result	Reference range
Haemoglobin	155 g/L	130–170 g/L
Haematocrit	45%	40–50%
WCC	8.9 × 10⁹/L	4–10 × 10⁹/L
Neutrophils	6.36 × 10⁹/L	1.5–6.5 × 10⁹/L
Platelets	263 × 10⁹/L	150–400 × 10⁹/L
MCV	91 fL	83–101 fL
CRP	<1 mg/L	<5 mg/L
Sodium	134 mmol/L	133–146 mmol/L
Potassium	4.7 mmol/L	3.5–5.1 mmol/L
Urea	6.0 mg/dL	7–20 mg/dL
Creatinine	67 mg/dL	59–101 mg/dL
eGFR	>90 mL/minute	>90 mL/minute
Total protein	67 g/L	63–82 g/L
Albumin	44 g/L	35–50 g/L
Corrected calcium	2.32 mmol/L	2.25–2.65 mmol/L
Bilirubin	15 μmol/L	3–22 μmol/L
ALP	49 IU/L	38–126 IU/L
ALT	18 IU/L	9–52 IU/L
APTT	20 seconds	22–30 seconds
INR	1.0	0.8–1.2
Random plasma glucose	6.0 mmol/L	4–11 mmol/L

**Figure 1:**
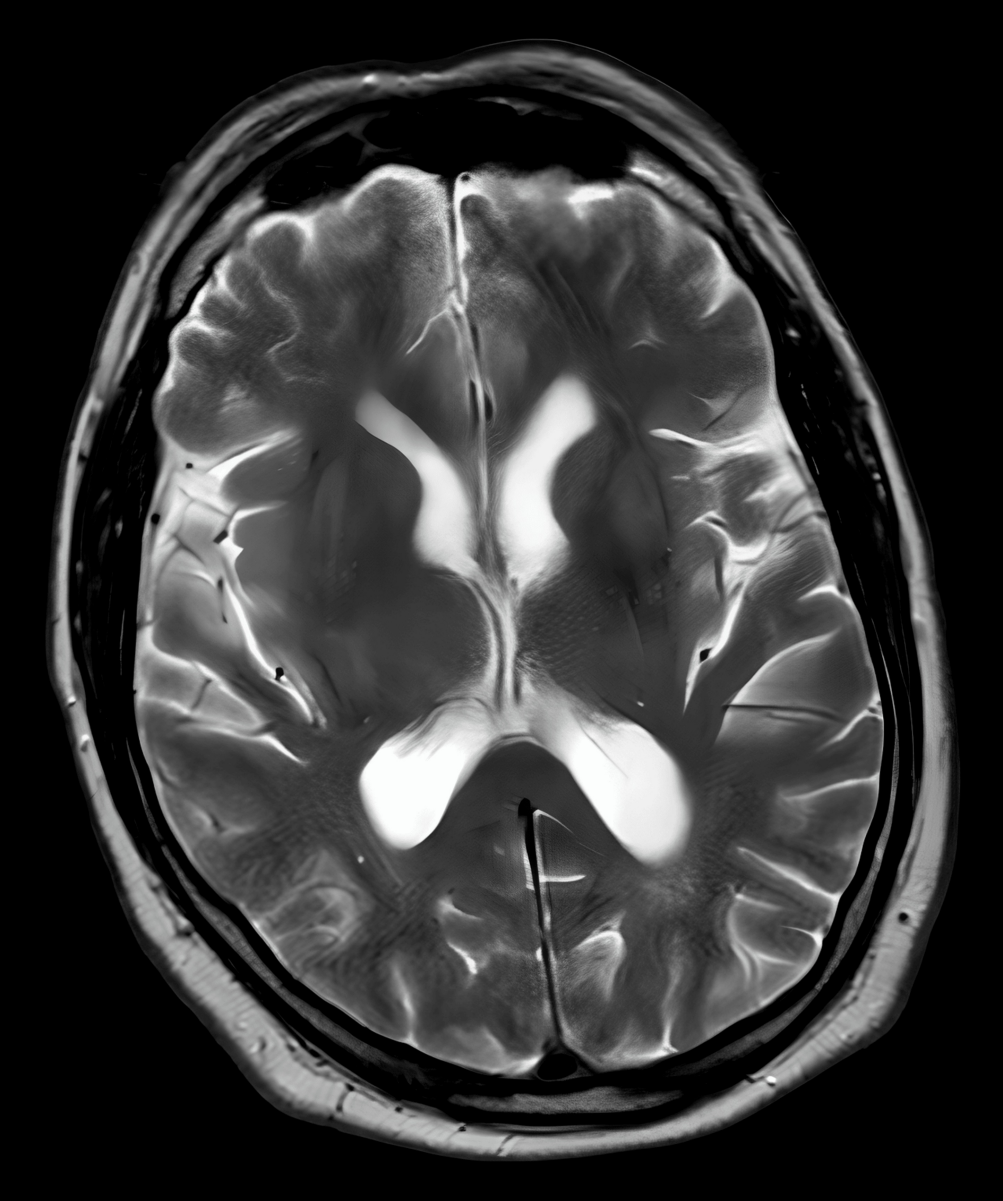
MRI of the head using a T2-weighted fluid-attenuated inversion recovery sequence, demonstrating normal brain parenchyma with no abnormal signal changes.

**Figure 2:**
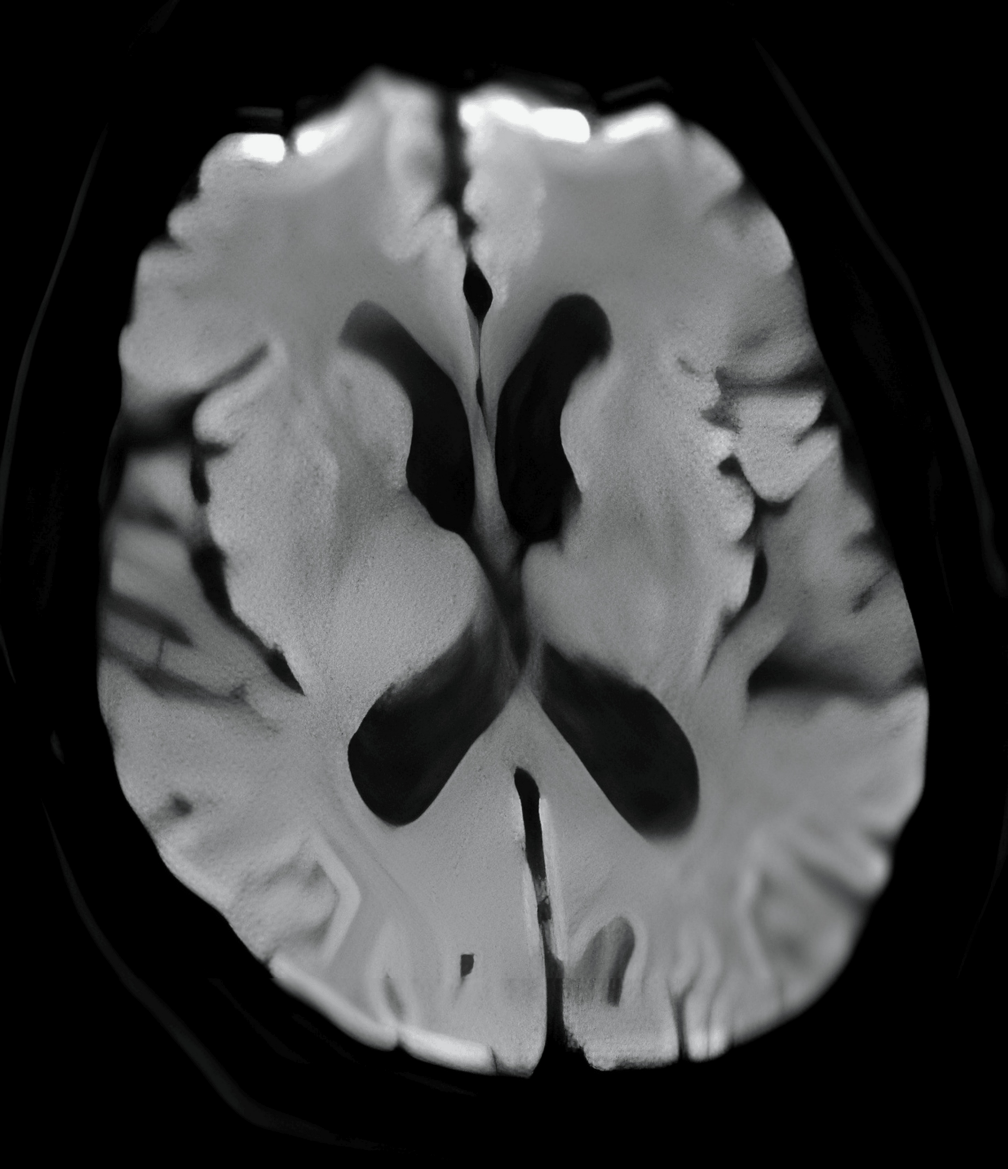
MRI of the head using a diffusion-weighted imaging sequence, demonstrating no evidence of restricted diffusion or acute infarction.

**Figure 3:**
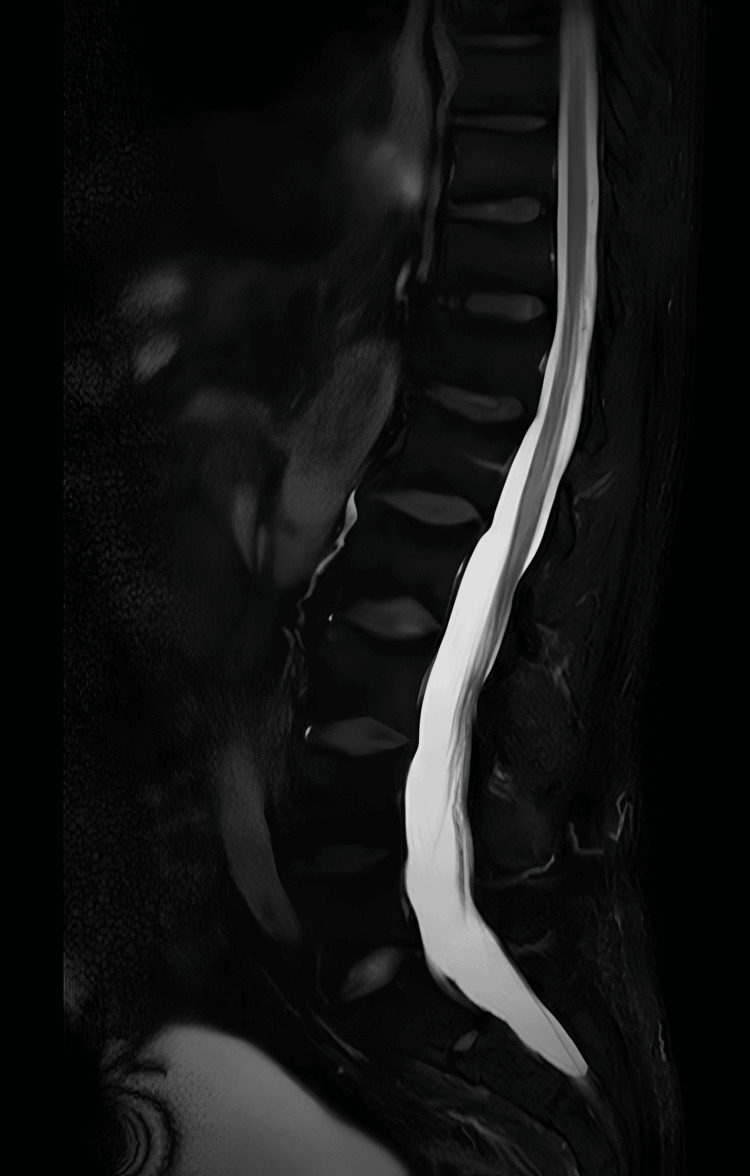
MRI of the entire spine (T2-weighted fluid-attenuated inversion recovery sequence; lower section shown), demonstrating preserved vertebral body height, alignment, and marrow signal. Mild multilevel disc desiccation is noted, with no evidence of significant disc prolapse, nerve root compression, canal stenosis, spinal cord compromise, or cauda equina enhancement.

**Figure 4:**
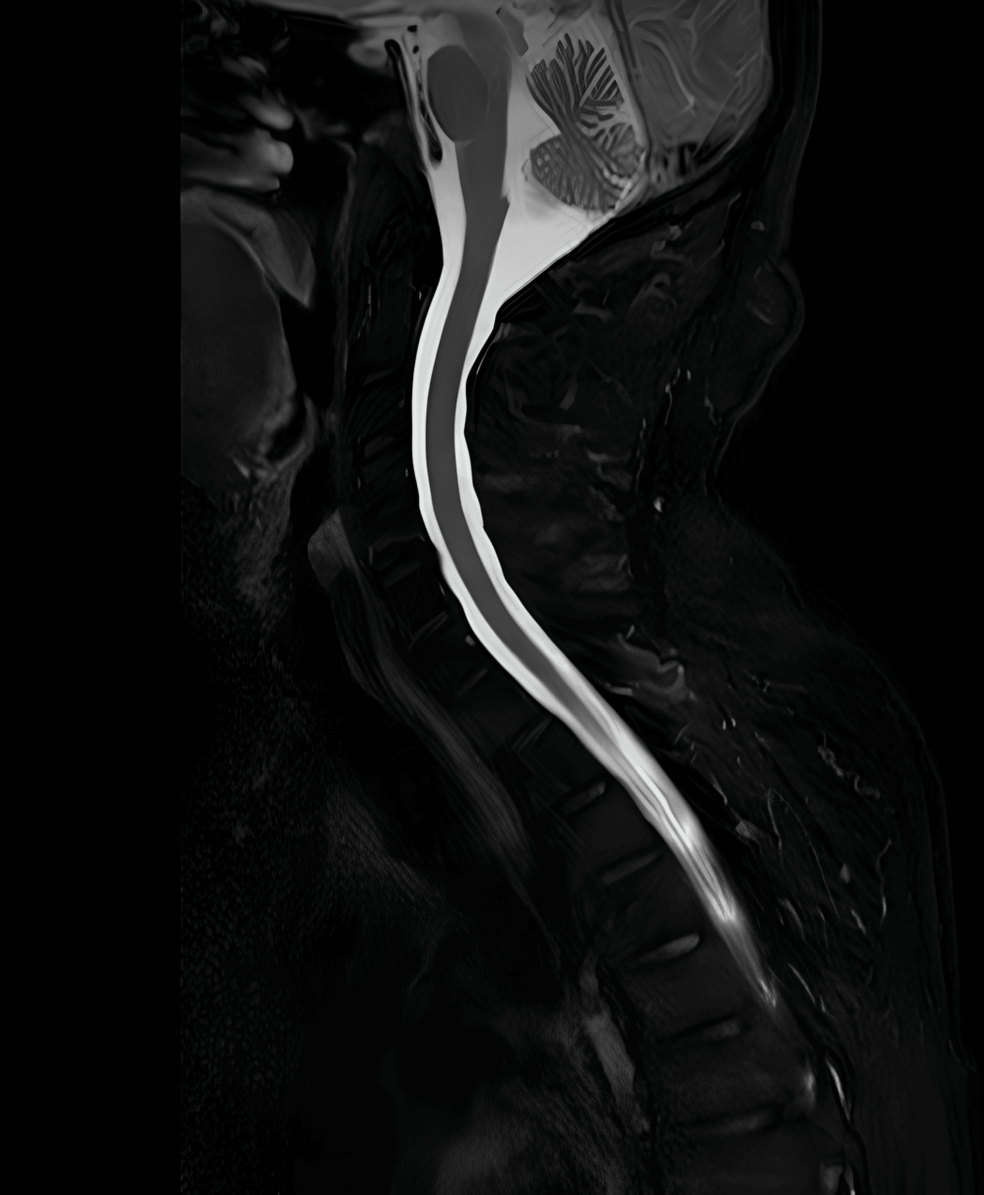
MRI of the entire spine (T2-weighted fluid-attenuated inversion recovery sequence; upper section shown), demonstrating preserved vertebral body height, alignment, and marrow signal, with no evidence of disc pathology, spinal canal stenosis, cord compression, or abnormal enhancement.

Neuroimaging was undertaken to exclude central causes of his symptoms. MRI of the head excluded acute infarct, demyelinating lesions, haemorrhage, and space-occupying lesions. MRI of the entire spine showed preserved vertebral body height, alignment, and marrow signal, with mild multilevel disc desiccation but no evidence of significant disc prolapses, nerve root compression, canal stenosis, spinal cord compromise, or cauda equina enhancement (Figures 1-4).

Given the absence of acute findings on imaging, a lumbar puncture was performed. Cerebrospinal fluid (CSF) analysis demonstrated albumin-cytological dissociation, characterised by an elevated protein level of 2.17 g/L (reference: 0.15-0.45 g/L) with a white cell count of 2 cells/µL (0-5 cells/µL) and no organisms identified on culture. The sample appeared clear and colourless, and oligoclonal band testing was negative in both serum and CSF. These findings supported a diagnosis of an acute demyelinating process, prompting further neurophysiological investigations. The summary of CSF analysis is shown in Table 3.

**Table 3:** Cerebrospinal fluid analysis reports. CSF = cerebrospinal fluid; WCC = white cell count; RBC = red blood cell

Trait	Result	Reference range
Appearance	Clear and colourless	Clear
CSF protein	2.17 g/L	0.15–0.45 g/L
CSF glucose	3.1 mmol/L	2.5–3.5 mmol/L
CSF WCC	2/µL	0–5/µL
CSF RBC	17/µL	0–5/µL
CSF culture	No growth	—
CSF oligoclonal bands	Negative	Negative

Therefore, nerve conduction studies were undertaken as advised by the neurology team. The results showed uniform slowing of motor conduction velocities and absent sensory responses consistent with longstanding CMT disease. However, new features were observed, including variability in distal motor latencies, temporal dispersion, and amplitude drop with proximal stimulation. These changes were interpreted as evidence of a superimposed demyelinating process, supporting the diagnosis of GBS. Ganglioside antibody testing was negative. The nerve conduction findings are illustrated in Tables 4-6.

**Table 4:** Nerve conduction study (motor summary table). Findings are consistent with demyelinating polyneuropathy. NR = no response; ms = milliseconds; O-P Amp = onset-to-peak amplitude; mV = millivolt; Site1/Site2 = stimulation and recording sites; Delta-0 = latency difference between two sites (ms); Dist = distance between stimulation sites (cm); Vel = conduction velocity (m/s); CTS = carpal tunnel syndrome; APB = abductor pollicis brevis; ADM = abductor digiti minimi; Uln = ulnar; A Elbow = above elbow; Med = median; Fib Head = fibular head; Poplit = popliteal fossa; Peroneal TA = peroneal motor to tibialis anterior; Tib Ant = tibialis anterior; Tibial Motor (Abd Hall Brev) = tibial nerve motor study recording over abductor hallucis brevis

Stimulation site	NR	Onset (ms)	O-P Amp (mV)	Site1	Site2	Delta-0 (ms)	Dist (cm)	Vel (m/s)
Bilateral CTS screening motor (APB/ADM)								
R Med Wrist		15.2	3.3	R Med Wrist	R Med Elbow	10.9	27.0	25
R Med Elbow		26.1	1.2	L Med Wrist	L Med Elbow	9.5	25.5	27
L Med Wrist		21.7	3.2	R Uln Wrist	R Uln A Elbow	15.1	33.5	22
L Med Elbow		31.2	1.6	L Uln Wrist	L Uln A Elbow	13.4	33.5	25
R Uln Wrist		11.0	2.4	-	-	-	-	-
R Uln A Elbow		26.1	0.8	-	-	-	-	-
L Uln Wrist		8.1	2.1	-	-	-	-	-
L Uln A Elbow		21.5	1.6	-	-	-	-	-
Left peroneal TA motor (Tib Ant)								
Fib Head		9.1	1.3	Poplit	Fib Head	4.4	9.0	20
Poplit		13.5	1.2	-	-	-	-	-
Left tibial motor (Abd Hall Brev)								
Ankle	NR	-	-	-	-	-	-	-

**Table 5:** Nerve conduction study (sensory summary table). Findings are consistent with demyelinating polyneuropathy. NR = no response; Onset (ms) = latency to onset of response in milliseconds; Peak (ms) = latency to peak in milliseconds; P-T Amp = peak-to-trough amplitude (µV); Site1/Site2 = stimulation and recording sites; Delta-0 (ms) = latency difference between two sites in milliseconds; Dist = distance between stimulation sites (cm); Vel = conduction velocity (m/s); Norm Vel = normal conduction velocity (m/s); L Sural = left sural nerve; Ankle = recording site at the ankle

Stimulation site	NR	Onset (ms)	Peak (ms)	P-T AmP (µV)	Site1	Site2	Delta-0 (ms)	Dist (cm)	Vel (m/s)	Norm Vel (m/s)
Left lower limb sensory (Ankle)										
L Sural	NR	-	-	-	L Sural	Ankle	-	0.0	-	-

**Table 6:** Nerve conduction study (ortho sensory summary table). Findings are consistent with demyelinating polyneuropathy. NR = no response; Onset (ms) = latency to onset of sensory potential in milliseconds; Peak (ms) = latency to peak in milliseconds; P-T Amp (µV) = peak-to-trough amplitude of sensory response in microvolts; Site1/Site2 = stimulation and recording sites; Delta-0 (ms) = latency difference between stimulation and recording sites in milliseconds; Vel (m/s) = conduction velocity in meters per second; L Med D2 = left median nerve, Digit 2; L Uln D5 = left ulnar nerve, Digit 5; R Rad = right radial nerve; L Rad = left radial nerve; Wrist = recording site at the wrist

Stimulation site	NR	Onset (ms)	Peak (ms)	P-T AmP (µV)	Site1	Site2	Delta-0 (ms)	Vel(m/s)
Bilateral digital ortho sensory (wrist)								
L Med D2	NR	-	-	-	L Med D2	Wrist	-	-
L Uln D5	NR	-	-	-	L Uln D5	Wrist	-	-
R Rad	-	2.8	3.0	0.0	R Rad	Wrist	2.8	-
L Rad	NR	-	-	-	L Rad	Wrist	-	-

This patient met several core diagnostic criteria for GBS, including a subacute onset of progressive, predominantly motor weakness, albumin-cytologic dissociation on CSF analysis (elevated protein at 2.17 g/L with normal white cell count), and neurophysiological evidence of a superimposed demyelinating process. While nerve conduction studies demonstrated longstanding uniform slowing consistent with CMT1A, new features, such as increased temporal dispersion, amplitude drop with proximal stimulation, and variability in distal latencies, indicated acute demyelination beyond baseline expectations. For example, median motor conduction velocities dropped to 22-27 m/second (versus expected >30 m/second in stable CMT1A), and distal latencies were prolonged. These findings, combined with the clinical context and exclusion of structural or metabolic causes, supported a diagnosis of acute GBS superimposed on chronic inherited neuropathy. Clinicians should be alert to the sudden progression of weakness, evolving electrophysiological patterns, and CSF changes in patients with known CMT, as this constellation strongly suggests an acute immune-mediated process such as GBS.

## Discussion

GBS is an acute, immune-mediated polyradiculoneuropathy, typically presenting with symmetrical, ascending motor weakness, areflexia, and variable sensory and autonomic involvement [10]. It is often preceded by infection, with *Campylobacter jejuni*, Epstein-Barr virus, cytomegalovirus, *Mycoplasma pneumoniae*, and SARS-CoV-2 being among the most frequently implicated pathogens [11]. Approximately one-third of cases remain idiopathic [12]. Although the typical phenotype is symmetrical and bilateral, asymmetrical presentations are reported in fewer than 1% of cases and pose a significant diagnostic challenge.

CMT1A, by contrast, is the most common hereditary motor and sensory neuropathy, caused by a duplication of the *PMP22* gene on chromosome 17. This leads to uniform demyelination of peripheral nerves, manifesting as slowly progressive distal weakness, muscle atrophy, pes cavus, sensory loss, and areflexia [13]. Nerve conduction studies in CMT1A reveal uniformly reduced conduction velocities and absent sensory responses.

The overlap of these two conditions is exceedingly rare. When they do co-occur, the clinical picture is complicated by overlapping signs, particularly absent reflexes and sensory impairment, which are features common to both conditions. In our patient, this challenge was compounded by the development of acute, progressive, unilateral lower limb weakness. At the time of presentation, he retained full strength in the left lower limb while the right deteriorated rapidly over 48 hours, an atypical course for classical GBS. Furthermore, there was no preceding infection, autonomic instability, or respiratory compromise.

From a diagnostic perspective, the absence of ascending weakness and bilateral involvement initially confounded clinical suspicion of GBS. Imaging ruled out structural causes, including myelopathy, radiculopathy, and cerebrovascular events. However, CSF analysis revealed albumin-cytological dissociation, and subsequent nerve conduction studies demonstrated findings not consistent with longstanding CMT alone. Specifically, the presence of variable distal motor latencies, temporal dispersion, and conduction block indicated an acute demyelinating process superimposed on chronic neuropathy, thus confirming the diagnosis of GBS.

The pathophysiological link between GBS and CMT1A is poorly understood [14], but emerging evidence suggests that inherited demyelinating disorders may predispose peripheral nerves to heightened vulnerability. Experimental models suggest that the overexpression of PMP22 in CMT1A alters Schwann cell-axon interaction and myelin integrity, which may, in turn, render peripheral nerves more susceptible to autoimmune attack [15]. In the context of GBS, molecular mimicry between microbial antigens and nerve components triggers an autoimmune response against myelin or axolemmal gangliosides, such as GM1 or GD1a [15]. While ganglioside antibodies were negative in our patient, the immunopathogenesis of GBS is heterogeneous, and seronegativity does not exclude the diagnosis.

This case highlights several clinically significant points. First, GBS can present atypically with asymmetrical, non-ascending weakness, particularly in patients with pre-existing neuropathies. Second, the co-existence of CMT1A may mask or mimic signs of acute deterioration, potentially delaying recognition of GBS. Third, interpretation of neurophysiological studies in such patients requires caution, as chronic demyelination from CMT can obscure acute changes unless carefully compared with previous studies or evaluated by an experienced neurophysiologist.

Importantly, this case reinforces the principle that early investigation with CSF analysis and neurophysiological testing should be pursued in cases of rapidly evolving neurological deficits, even when classical features of GBS are absent. The patient’s favourable response to IVIG, in the absence of respiratory or bulbar involvement, supports current guideline recommendations for the timely initiation of immunotherapy in clinically compatible scenarios.

There is limited literature exploring the asymmetrical GBS presentations with CMT1A. In one instance, there was no identifiable infectious trigger; in others, preceding illnesses included pneumonia and gastroenteritis [13]. Although the pathogenesis of unilateral weakness in GBS remains poorly defined, anti-ganglioside antibodies, particularly anti-GM1 and anti-GD1a, have been implicated in some cases. A previous study described a rare case of asymmetrical GBS associated with anti-GD1a antibodies following gastroenteritis; however, other reports have documented asymmetry in the absence of such serological markers. This variability suggests a heterogeneous immunological basis and highlights the limitations of relying solely on antibody testing for diagnostic confirmation [15].

Management of GBS remains largely supportive, encompassing regular bowel and bladder care, venous thromboembolism prophylaxis, serial assessment of respiratory function (e.g. forced vital capacity), pain control, and swallowing evaluation where indicated [16]. Immunomodulatory therapy is warranted in cases with progressive weakness, and both IVIG and plasma exchange have demonstrated equivalent efficacy. In our case, the decision to initiate IVIG was made in light of the patient’s clinical deterioration and background neurological deficits, including CMT1A and prior cerebrovascular disease. Treatment was well-tolerated, with gradual improvement in motor function and no complications. The patient remains under ongoing follow-up with neurology services.

The patient was managed with a multidisciplinary approach involving neurology, stroke medicine, physiotherapy, occupational therapy, and the acute medical team. Analgesia was initiated for symptomatic relief of lumbar pain, comprising regular paracetamol 1 g four times daily, ibuprofen 400 mg three times daily, and morphine 10 mg as required. Despite adequate pain control, his functional capacity deteriorated, and he became increasingly dependent in activities of daily living.

In light of the clinical progression and supportive findings from CSF analysis and nerve conduction studies, IVIG was commenced at a dose of 0.4 g/kg/day for five consecutive days, in accordance with neurology team recommendations. Respiratory function was monitored routinely throughout admission using peak expiratory flow measurements, which remained within his baseline range, and there were no signs of respiratory compromise. Physiotherapy and occupational therapy input were provided daily with a focus on strength recovery and mobility rehabilitation. These interventions supported the patient in regaining muscle function and preventing complications associated with immobility.

Although initial plans were made for review at one month post-discharge, the patient was followed up after two months due to service delays. At that point, he had made a marked recovery. Motor function had returned to baseline, with MRC grade 5/5 power in both lower limbs. He reported ongoing but manageable low back discomfort and intermittent paraesthesia; however, he was functionally independent, able to mobilise up to two miles, and no longer required assistance with daily activities. His sleep, appetite, and bowel and bladder functions were reported as normal. Repeat nerve conduction studies demonstrated some improvement in distal motor latencies, consistent with partial recovery of the superimposed acute neuropathy. Nevertheless, residual abnormalities persisted, in keeping with his underlying diagnosis of CMT disease type 1A. He remains under outpatient neurology surveillance for long-term monitoring and support. The patient is alive and stable at the time of writing this case report.

## Conclusions

This case report adds to the limited literature on coexistent GBS and CMT1A and underscores the importance of considering acute demyelinating neuropathies in patients with chronic neurological conditions, particularly when new or rapidly progressive weakness arises. The diagnosis in this case was supported by the sudden onset of asymmetrical lower limb weakness, albuminocytologic dissociation on CSF analysis, and new conduction block and temporal dispersion on nerve conduction studies, findings not typical of stable CMT1A. The patient’s improvement following IVIG therapy further supports an acute immune-mediated process. Clinicians should maintain a high index of suspicion and initiate appropriate diagnostic workup without relying solely on typical GBS presentations. In practice, any abrupt clinical deterioration in patients with hereditary neuropathy should prompt consideration of a superimposed condition such as GBS, as early recognition and treatment can significantly improve outcomes.
